# *Rickettsia japonica* Infection after Land Leech Bite, Japan

**DOI:** 10.3201/eid2506.181985

**Published:** 2019-06

**Authors:** Eiichiro Sando, Motoi Suzuki, Mitsuya Katayama, Masakatsu Taira, Hiromi Fujita, Koya Ariyoshi

**Affiliations:** Kameda Medical Center, Kamogawa, Japan (E. Sando, M. Katayama);; Nagasaki University, Nagasaki, Japan (E. Sando, M. Suzuki, K. Ariyoshi);; Chiba Prefectural Institute of Public Health, Chiba, Japan (M. Taira); Mahara Institute of Medical Acarology, Anan, Japan (H. Fujita)

**Keywords:** Japanese spotted fever, Rickettsia, scrub typhus, Orientia tsutsugamushi, leech, Rickettsia japonica, bacteria, zoonoses, Japan

## Abstract

We report a case of *Rickettsia japonica* infection in an 81-year-old man in central Japan. The patient had fever, rash, and an eschar but no evidence of a tick bite. His symptoms began 8 days after a land leech bite. The land leech is a potential vector of *R. japonica*.

Japanese spotted fever, a tickborne disease caused by *Rickettsia japonica*, has been reported in Japan, Korea, and China (*1**–**3*). We describe a case of Japanese spotted fever after a land leech (*Haemadipsa zeylanica japonica*) bite.

On August 3, 2016, an 81-year-old man was transported to an emergency department with a 2-day history of fever (temperature of 38°C), staggering, appetite loss, and general malaise. He was undergoing hormonal therapy for prostate cancer and had an indwelling urinary catheter. However, he was fully independent and walked 2 km every day as a tour guide to a mountain road in the southern Boso area of Japan. At admission, he was alert and oriented, with no apparent fever (temperature of 36.8°C). Clinicians observed a nonpruritic, painless rash on his torso and extremities (Figure, panels A, B), including his palms and the soles of his feet. The attending physician thoroughly searched for an eschar and noted only a single nonpruritic, painless lesion on the man’s lower abdomen (Figure, panel C). The patient and his family reported that the eschar appeared at the site where a land leech had been attached on July 24, 10 days before admission, and that the site bled when the leech was removed. They denied any tick bite.

**Figure:**
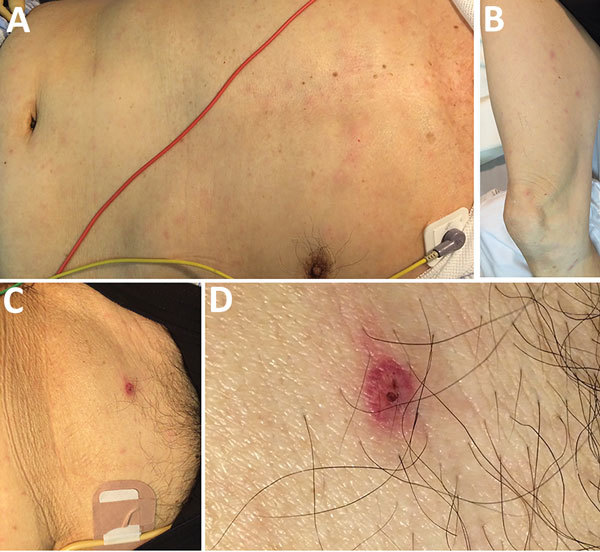
Patient with *Rickettsia japonica* infection after being bitten by a land leech, Japan. A, B) Erythematous macular rash on the patient’s torso (A) and extremities (B); C, D) eschar on the lower abdomen showing an atypical appearance with a relatively well-demarcated boundary of erythema with a tiny scab.

Notable laboratory data included low platelet count (102,000/µL), slightly elevated aspartate aminotransferase (52 IU/L), elevated lactate dehydrogenase (468 IU/L), and elevated C-reactive protein (5.37 mg/dL). Urinalysis was positive for protein and occult blood. Chest radiograph and electrocardiogram findings were unremarkable.

We suspected rickettsial disease because the patient had typical symptoms, including fever, rash, and eschar, and a history of walking in the mountains. We sent his blood samples and the crust of the eschar to the Chiba Prefectural Institute of Public Health (Chiba, Japan) for indirect immunofluorescence and PCR assays (Appendix). In addition, the blood samples were tested at the Mahara Institute of Medical Acarology (Anan, Japan) with indirect immunoperoxidase assay to identify 6 *Orientia tsutsugamushi* serotypes: Kato, Karp, Gilliam, Irie/Kawasaki, Hirano/Kuroki, and Shimokoshi.

The patient received 100 mg of minocycline intravenously every 12 hours for 7 days and received the same dose orally for 1 day after he was discharged. His symptoms promptly resolved without any complications. 

Paired serum antibody titers against *R. japonica* in the acute phase (day 1 of treatment) were IgM <1:20 and IgG <1:20 but increased to IgM 1:1,280 and IgG 1:10,240 in the convalescent phase (day 21 of treatment). The samples tested for *O. tsutsugamushi* were negative for all serology except IgG titer against serotypes Karp (1:160) and Hirano/Kuroki (1:80) in both acute and convalescent phases, indicating past infection with *O. tsutsugamushi*. Target genes obtained from the eschar were identical with *R. japonica*; the 17-kDa protein had 100% sequence homology and *gltA* 99.5% (Appendix Figures 1, 2).

We detected *R. japonica* from the eschar formed after a land leech bite in a patient without evidence of a tick bite. Most patients with rickettsial diseases, such as Japanese spotted fever and scrub typhus, do not notice a tick or mite bite (*4*), but a leech bite is easy to detect because the site bleeds for an extended time due to hirudin in leech saliva. We conducted a thorough physical examination to check for tick bites but found no additional eschar on this patient. In our experience (*4*), a typical eschar caused by a tick or mite bite appears as a circular crater with a scab, red flare with an indistinct border, and desquamation. However, the eschar in this case was atypical because of a relatively well-demarcated boundary of erythema with a tiny scab, (Figure, panel D).

A new species of *Rickettsia* was detected from leeches in Japan (*5*,*6*). Furthermore, certain leech species, parasitizing frogs or fish, can complete the vertical transmission of *Rickettsia* spp. with possible horizontal transmission (*6*). The leech is reported to be a potential vector for human rickettsial infections (*7*,*8*). Slesak et al. described the case of a 39-year-old woman with *R. felis* infection confirmed by eschar PCR after a leech bite in northern Laos (*7*). Balcells et al. reported the case of a 54-year-old man with scrub typhus–like illness after a leech bite in southern Chile (*8*). In our previous study (*4*), 13% (4/31) of patients with Japanese spotted fever and 2% (4/188) of patients with scrub typhus diagnosed by serologic tests had a history of land leech bite before the symptom onset.

Our report is limited because we did not have the land leech for testing by PCR. The patient might have had rickettsia on his skin and then been inoculated by the leech bite or by scratching after the bite (*7*). Further investigations, including an experimental model, are needed to support the potential role of leeches in the transmission of *R. japonica* and other *Rickettsia* spp.

Appendix PCR assay, DNA sequencing, and phylogenetic trees of *Rickettsia japonica* in patient with land leech bite, Japan.
