# Supplementary material for: Rickettsia japonica Infection after Land Leech Bite, Japan
Source: Emerg Infect Dis. 2019 Jun;25(6):1243–5. doi: 10.3201/eid2506.181985 (PMC6537741; doi:10.3201/eid2506.181985)
Supplement: Appendix — PCR assay, DNA sequencing, and phylogenetic trees of Rickettsia japonica in patient with land leech bite, Japan. [file 18-1985-Techapp-s1.pdf]

Article DOI: https://doi.org/10.3201/eid2506.181985

Rickettsia japonica Infection after Land Leech Bite

Appendix

Polymerase chain reaction (PCR) assay and DNA sequencing

We sent an eschar sample collected from the patient to the Chiba Prefectural Institute of Public Health (Chiba, Japan). DNA was extracted from the sample by using the High Pure PCR Preparation kit (Roche Diagnostics, https://www.roche.com) according to the manufacturer's instructions. A duplex real-time PCR assay targeting a fragment of the 16S rDNA was performed for the simultaneous detection of *O. tsutsugamushi* and spotted fever group *Rickettsia* spp. (*1*).

Following the detection of spotted fever group *Rickettsia* spp. DNA, the 17 kDa protein and *glt*A genes were amplified (*2*,*3*). Distilled water was used as a negative control and *R. japonica* YH strain as a positive control. The PCR amplicons were further sequenced by using BigDye Terminator v1.1 Cycle Sequencing Kit and Applied Biosystems 3130 Genetic Analyzer (ThermoFisher Scientific, https://www.thermofisher.com). Phylogenetic trees were constructed in MEGA 6.0 software (https://www.megasoftware.net) using the neighbor-joining method. Bootstrap values were calculated based on 1,000 replications of the alignment (*4*).

The target genes from the patient's eschar were submitted to GenBank, accession number LC460478 for 17 kDa protein, and accession number LC460479 for *gltA* gene.

References

1. Kawamori F, Shimazu Y, Sato H, Monma N, Ikegaya A, Yamamoto S, et al. Evaluation of diagnostic assay for rickettsioses using duplex real-time PCR in multiple laboratories in Japan. Jpn J Infect Dis. 2018;71:267–73. PubMed http://dx.doi.org/10.7883/yoken.JJID.2017.447

2. Mediannikov OY, Sidelnikov Y, Ivanov L, Mokretsova E, Fournier PE, Tarasevich I, et al. Acute tick-borne rickettsiosis caused by *Rickettsia heilongjiangensis* in Russian Far East. Emerg Infect Dis. 2004;10:810–7. PubMed http://dx.doi.org/10.3201/eid1005.030437

3. Furuya Y, Katayama T, Yoshida Y, Kaiho I. Specific amplification of *Rickettsia japonica* DNA from clinical specimens by PCR. J Clin Microbiol. 1995;33:487–9. PubMed

4. Tamura K, Stecher G, Peterson D, Filipski A, Kumar S. MEGA6: Molecular Evolutionary Genetics Analysis version 6.0. Mol Biol Evol. 2013;30:2725–9. PubMed http://dx.doi.org/10.1093/molbev/mst197

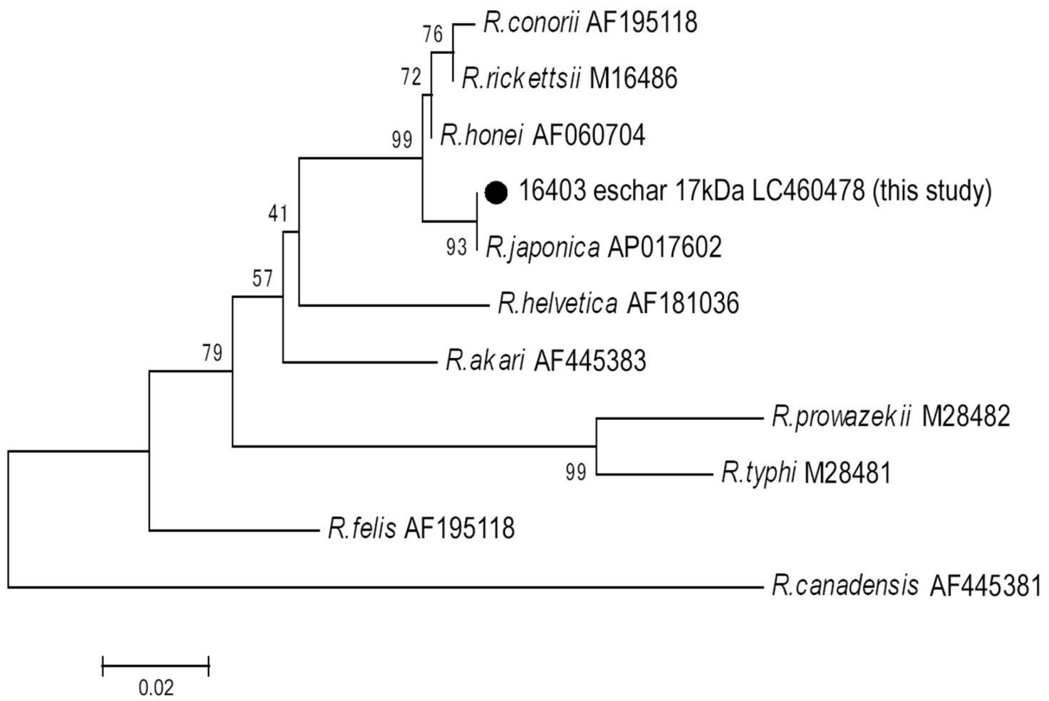

Appendix Figure 1. Phylogenetic tree of *Rickettsia* spp. genes comparing 17 kDa protein from eschar of a land leech bite, Japan. Phylogenetic relationships based on the sequence of the 357-bp fragment of the omp gene for 17 kDa protein. Circle represents sequence from this study. Scale bar indicates nucleotide substitutes per site.

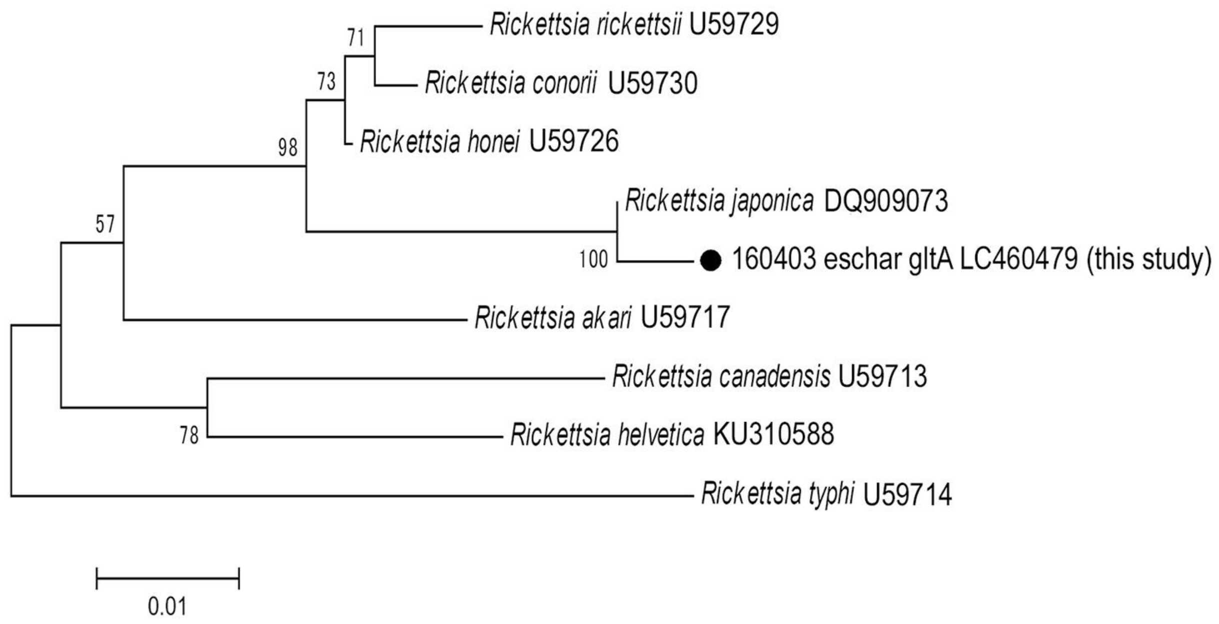

Appendix Figure 2. Phylogenetic tree of *Rickettsia* spp. genes comparing *gltA* from eschar of a land leech bite, Japan. Phylogenetic relationships based on the sequence of the 388-bp fragment of the *gltA* gene for citrate synthase. Scale bar indicates nucleotide substitutes per site.
